# Varicella-zoster virus recapitulates its immune evasive behaviour in matured hiPSC-derived neurospheroids

**DOI:** 10.3389/fimmu.2024.1458967

**Published:** 2024-09-16

**Authors:** Jonas Govaerts, Elise Van Breedam, Sarah De Beuckeleer, Charlotte Goethals, Claudio Peter D’Incal, Julia Di Stefano, Siebe Van Calster, Tamariche Buyle-Huybrecht, Marlies Boeren, Hans De Reu, Søren R. Paludan, Marc Thiry, Marielle Lebrun, Catherine Sadzot-Delvaux, Helena Motaln, Boris Rogelj, Johan Van Weyenbergh, Winnok H. De Vos, Wim Vanden Berghe, Benson Ogunjimi, Peter Delputte, Peter Ponsaerts

**Affiliations:** ^1^ Laboratory of Experimental Hematology (LEH), Vaccine and Infectious Disease Institute (Vaxinfectio), University of Antwerp, Antwerp, Belgium; ^2^ Laboratory of Microbiology, Parasitology and Hygiene (LMPH), University of Antwerp, Antwerp, Belgium; ^3^ Antwerp Center for Translational Immunology and Virology (ACTIV), Vaccine and Infectious Disease Institute (Vaxinfectio), University of Antwerp, Antwerp, Belgium; ^4^ Laboratory of Cell Biology and Histology, Antwerp Center for Advanced Microscopy, Department of Veterinary Sciences, University of Antwerp, Wilrijk, Belgium; ^5^ µNEURO Research Centre of Excellence, University of Antwerp, Wilrijk, Belgium; ^6^ Cell Death Signaling – Epigenetics Lab, Department of Biomedical Sciences, University of Antwerp, Antwerp, Belgium; ^7^ Flow Cytometry and Cell Sorting Core Facility (FACSUA), University of Antwerp, Antwerp, Belgium; ^8^ Department of Biomedicine, Aarhus University, Aarhus, Denmark; ^9^ Laboratory of Cell and Tissue Biology, GIGA-Neurosciences, Cell Biology L3, University of Liège, Liege, Belgium; ^10^ Laboratory of Virology and Immunology, GIGA-Infection, Inflammation and Immunity, University of Liège, Liège, Belgium; ^11^ Department of Biotechnology, Jozef Stefan Institute, Ljubljana, Slovenia; ^12^ Faculty of Chemistry and Chemical Technology, University of Ljubljana, Ljubljana, Slovenia; ^13^ Department of Microbiology, Immunology and Transplantation, Laboratory of Clinical and Epidemiological Virology, Rega Institute, KU Leuven, Leuven, Belgium; ^14^ Antwerp Unit for Data Analysis and Computation in Immunology and Sequencing (AUDACIS), Antwerp, Belgium; ^15^ Centre for Health Economics Research and Modelling Infectious Diseases (CHERMID), Vaccine and Infectious Disease Institute (Vaxinfectio), University of Antwerp, Antwerp, Belgium; ^16^ Department of Paediatrics, Antwerp University Hospital, Antwerp, Belgium; ^17^ Infla-Med, University of Antwerp, Antwerp, Belgium

**Keywords:** human iPSc, neurospheroids, varicella-zoster virus, type-I interferon signalling, antigen presentation, stress granules, structural integrity

## Abstract

Varicella-zoster virus (VZV) encephalitis and meningitis are potential central nervous system (CNS) complications following primary VZV infection or reactivation. With Type-I interferon (IFN) signalling being an important first line cellular defence mechanism against VZV infection by the peripheral tissues, we here investigated the triggering of innate immune responses in a human neural-like environment. For this, we established and characterised 5-month matured hiPSC-derived neurospheroids (NSPHs) containing neurons and astrocytes. Subsequently, NSPHs were infected with reporter strains of VZV (VZV^eGFP-ORF23^) or Sendai virus (SeV^eGFP^), with the latter serving as an immune-activating positive control. Live cell and immunocytochemical analyses demonstrated VZV^eGFP-ORF23^ infection throughout the NSPHs, while SeV^eGFP^ infection was limited to the outer NSPH border. Next, NanoString digital transcriptomics revealed that SeV^eGFP^-infected NSPHs activated a clear Type-I IFN response, while this was not the case in VZV^eGFP-ORF23^-infected NSPHs. Moreover, the latter displayed a strong suppression of genes related to IFN signalling and antigen presentation, as further demonstrated by suppression of IL-6 and CXCL10 production, failure to upregulate Type-I IFN activated anti-viral proteins (Mx1, IFIT2 and ISG15), as well as reduced expression of CD74, a key-protein in the MHC class II antigen presentation pathway. Finally, even though VZV^eGFP-ORF23^-infection seems to be immunologically ignored in NSPHs, its presence does result in the formation of stress granules upon long-term infection, as well as disruption of cellular integrity within the infected NSPHs. Concluding, in this study we demonstrate that 5-month matured hiPSC-derived NSPHs display functional innate immune reactivity towards SeV infection, and have the capacity to recapitulate the strong immune evasive behaviour towards VZV.

## Graphical Abstract

Created with BioRender.com.
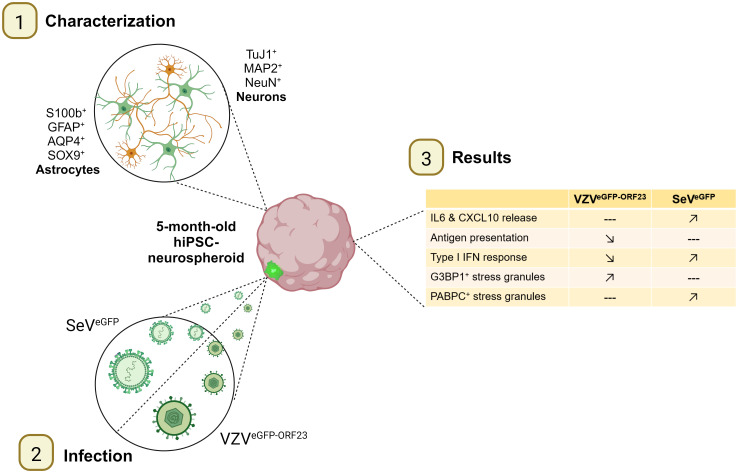

## Highlights

- Characterisation of 5-month-old hiPSC-derived neurospheroids (NSPHs).
- Varicella zoster virus and Sendai virus infection of NSPHs.
- Molecular and cellular profiling of innate immunity in NSPHs.
- Viral immune evasion and stress granule formation in NSPHs.

## 1 Introduction

Varicella-zoster virus (VZV) is a highly species-specific human neurotropic alphaherpesvirus that, due to its long co-evolutionary history with the human host, has developed a wide range of immune evasion mechanisms preventing early host immune activation (1, 2). Although VZV has a high transmission rate, resulting in the natural infection of most children before the age of 10 and the typical varicella (chickenpox) disease phenotype, complications can be unpredictable (3, 4). While infection is usually self-limiting, VZV remains in a latent state in the neurons of the sensory ganglia. Reactivation of VZV causes herpes zoster (HZ), which is characterised by a more localised, painful skin rash with blisters. In addition, about 20% of HZ patients suffer from debilitating, long-lasting pain known as postherpetic neuralgia (PHN) (5). Neurological complications in the central nervous system (CNS) can also occur (4). Here, the most common manifestation of VZV infection is vasculopathy, which manifests as headaches, cognitive decline and/or focal neurological deficits (6). In rare cases, VZV infection can lead to even more severe neurological complications such as meningitis, encephalitis, cerebellitis and myelopathy (4, 6). The most common hallmarks underlying these pathologies include persistent inflammation and/or virus-induced hypercoagulability (6). Although VZV-related CNS infections are treatable with intravenous acyclovir therapy and corticosteroids, the wide spectrum of possible CNS complications is not necessarily accompanied by a VZV-associated rash, potentially leading to mis- or non-diagnosis (6). Consequently, this may lead to long-lasting CNS dysfunction and emphasizes the need for a better understanding of VZV neuropathogenesis in the CNS. A specific focus on the neuro-immune mechanisms of disease and/or protection could advance both preventive and therapeutic strategies to avoid or efficiently cure severe neurological complications following VZV infection of CNS tissue.

Even though there is an unmet need for studying VZV neuropathology, its exclusive human tropism limits profound cellular studies due to the scarcity of human CNS tissue available for research. To cope with this, recent advances in cell and developmental biology have led to the establishment of self-organising, stem cell-derived 3D cultures containing a mixture of different CNS cell types (7, 8). These structures are often referred to as neurospheroids (NSPHs) or brain organoids, and have demonstrated improved differentiation capabilities, time-dependent maturation, and superior functionality of neural cell types as compared to classically used (stem) cell line-derived 2D cultures (9–12). In the context of viral infection, stem cell-derived brain organoids have already been applied to investigate neurodevelopmental and neuro-immune consequences for neurotropic viruses of significant public health concern, such as - but not limited to - Zika virus (ZIKV) (13, 14), herpes simplex virus 1 (HSV1) (13, 15), and more recently severe acute respiratory syndrome coronavirus 2 (SARS-CoV-2) (16–18). However, to our knowledge, no stem cell-derived NSPH models have been used to study VZV neuro-immune interactions (7, 19, 20). Continuing on previously reported 2D human pluripotent stem cell derived neuronal and neuro-immune co-culture models studying VZV infection (5, 21–25), in this study we aim to investigate whether a productive VZV infection is immunologically recognised within a neural-like NSPH environment.

This research question directly relates to our preceding studies whereby innate immune recognition of VZV was investigated in a 2D human (h)iPSC-derived compartmentalised neuronal model (25). Although hiPSC-derived neurons are able to adequately respond to IFNα signalling and suppress a productive VZV infection upon IFNα treatment, they do not mount a Type-I IFN response themselves upon VZV infection, and as such are unable to autonomously suppress a productive VZV infection (25). Subsequently, we hypothesised that co-culture of VZV-infected hiPSC-derived neurons with isogenic hiPSC-derived macrophages, as an immune-competent bystander population, could control a productive VZV infection. However, even though hiPSC-derived macrophages were demonstrated to be fully immune competent, no Type-I IFN response was mounted upon VZV challenge, and no suppression of productive VZV infection within co-cultured neurons was seen (5). Within the CNS, additional cells such as astrocytes and microglia play an important role in immune surveillance and may thus be able to control a productive VZV infection (26). Given the functional similarities between microglia and macrophages, and based on our preceding work, we do not expect microglia to be able to mount a strong Type-I IFN response upon VZV recognition. Therefore, in this study we focused on the potential role of astrocytes as bystander immune cells to control VZV infection.

Based on previous reports demonstrating that astrocyte maturation is highly essential for shifting their functional role from support in brain development to support in brain homeostasis and immunity (9, 11), we here opted to investigate cellular responsivity to VZV infection in 5-month matured hiPSC-derived NSPHs. Furthermore, infection of NSPHs with a murine Sendai virus, which is expected to trigger a strong Type-I IFN response, was applied as positive control to allow better understanding of the immune evasive behaviour of VZV in a human neural-like environment (25).

## 2 Materials and methods

### 2.1 NSPH generation, differentiation, and maturation

A previously established and characterised self-renewing hiPSC-derived neural stem cell line (hiPSC-NSC) was cultured as described in Van Breedam et al. (27). For the initial generation of NSPHs, hiPSC-NSCs were harvested and seeded at a density of 1.6 x 10^4^ cells per well in an ultra-low attachment (ULA) 96-well plate (Costar, 7007) in 100µL of complete neural expansion medium [cNEM, consisting of 1:1 Neurobasal medium (Gibco, 21103-049): Advanced DMEM/F12 (Gibco, 12634-010) supplemented with 1x neural induction supplement (Gibco, A16477-01) and 1% Penicillin/Streptomycin solution (Gibco, 15070-063)]. NSPH cultures were maintained in a humidified cell culture incubator at 37°C and 5% CO_2_. On the third day post-seeding, 100µL of cNEM was added and from this point onwards, the cultures were kept under constant orbital shaking at 88 rpm. Partial (50%) medium changes were then performed every 2-3 days until day 14. At this point, NSPHs were transferred to ULA 6-well plates (Corning, 3471) with 5-6 NSPHs per well in 3 mL cNEM. Partial (50%) medium changes were performed every 2-3 days throughout the culture period until the age of 5 months.

### 2.2 Propagation of VZV^eGFP-ORF23^ in ARPE19 cells

The human retinal pigment epithelial cell line ARPE19 (ATCC, CRL-2302) was used for the propagation of the VZV^eGFP-ORF23^ strain, as previously described (25, 28). In this pOka-derived recombinant VZV strain, the minor capsid protein ORF23 is fused with the enhanced green fluorescent protein (eGFP). Following VZV^eGFP-ORF23^ propagation in ARPE19 cells, infected cells were harvested and cryopreserved in 900µL cARPE19 medium [90% DMEM/F12 (Gibco, 11320-074) + 10% FBS (Gibco, 10270-106)] + 100µL DMSO (Sigma, D2650) at a concentration of 1.0 x 10^6^ cells per vial. After thawing, VZV^eGFP-ORF23^ titres in plaque-forming units (PFU) were determined using an infectious foci assay in ARPE19 cells, according to previously described procedures (25, 28, 29).

### 2.3 Generation and propagation of VZV^ORF65-tdT-66^ in ARPE19 cells

The VZV WT-tdTomato (VZV^ORF65-tdT-66^) strain, based on the pOka-derived recombinant VZV strain, was generated by cloning the tdTomato-tag in the intergenic region between ORF65 and 66 under the control of the SV40 early promotor, according to previously described procedures (28). This newly generated VZV^ORF65-tdT-66^ was maintained in ARPE19 cells as described above.

### 2.4 Generation of ARPE19-eGFP cells

ARPE19 cells stably expressing the eGFP-reporter protein were generated following transduction with a Lentiviral vector (LVv) encoding eGFP and the hygromycin resistance protein (pCHMWS-eGFP-IRES-Hyg^r^, kindly provided by the Leuven Viral Vector Core, LVVC, Molmed, KU Leuven, Leuven, Belgium) (25). Stable transduction and selection were performed as described previously (30). Stable expression of eGFP was confirmed by both fluorescence microscopy and flow cytometric analysis. ARPE19-eGFP cells were cryopreserved in 900µL cARPE19 medium + 100µL DMSO at a concentration of 1.0 x 10^6^ cells per vial.

### 2.5 Pro-inflammatory stimulation of NSPHs

For pro-inflammatory stimulation, NSPHs were treated with a combination of 27.5 µg/mL ATP (Avantor, ICNA0219461301), 1 µg/mL LPS (Sigma, L7895), 10ng/mL IL1β (Immunotools GmbH, 11340012), 50ng/mL IFNγ (Immunotools GmbH, 11343534) and 10ng/mL TNFα (Immunotools GmbH, 11343013) for 72 hours, after which the cell culture supernatant was collected and frozen at -80°C for subsequent analysis.

### 2.6 VZV^eGFP-ORF23^ and VZV^ORF65-tdT-66^ infection of NSPHs

For infection of NSPHs, VZV^eGFP-ORF23^-infected ARPE19 cells were thawed and used directly as a vehicle for cell-associated VZV^eGFP-ORF23^ infection. In this study, 3.25 x 10^3^ PFU were added per NSPH, corresponding to 16.25 x 10^3^ VZV^eGFP-ORF23^-infected ARPE19 cells. As negative control for VZV^eGFP-ORF23^ infection in downstream experiments, NSPHs were stimulated with ARPE19-eGFP cells. For this, an equal amount of 16.25 x 10^3^ ARPE19-eGFP cells were added to the NSPHs. Medium changes were performed at 3dpi (80%), 5dpi (50%) and 7dpi (50%), thereby removing VZV^eGFP-ORF23^-infected ARPE19 and ARPE19-eGFP cells. Supernatant collected at 3, 5 and 7dpi was centrifuged to remove debris and frozen at -80°C for downstream analysis. Similarly, VZV^ORF65-tdT-66^ infected ARPE19 cells were used directly as a vehicle for cell-associated VZV ^ORF65-tdT-66^ infection of NSPHs, as described above.

### 2.7 SeV^eGFP^ infection of NSPHs

Commercially available eGFP-labelled Sendai Virus (SeV^eGFP^) was obtained from ViraTree (S124). For NSPH infection, 3.25 x 10^3^ PFU were added per NSPH, with medium changes performed at 3dpi (80%), 5dpi (50%) and 7dpi (50%), thereby removing remaining free SeV^eGFP^. Supernatant collected at 3, 5 and 7dpi was frozen at -80°C for downstream analysis.

### 2.8 Single cell dissociation of NSPHs

NSPHs were dissociated into a single cell suspension using a commercially available Papain/DNase-I dissociation kit (Worthington, 9035-81-1) following a procedure described by Barbar et al., 2020 (31, 32), with minor modifications. Briefly, 2-4 NSPHs were transferred into a single 24-well and mechanically broken into smaller pieces using sterile tweezers. Next, Papain/DNase-I solution (1 mL) was added and NSPH pieces were gently triturated 3 times. Subsequently, NSPH pieces were incubated for 45 minutes on an orbital shaker (88 rpm at 37°C), gently triturated 10 times, re-incubated for 15 minutes under orbital shaking and gently triturated again 10 times. The NSPH dissociation process was then stopped by transferring the dissociated cell suspension to an ovomucoid inhibitor solution (1,2 mL + 2 mL Earle’s medium). Finally, cells were centrifuged and resuspended in PBS.

### 2.9 Flow cytometry analysis of NSPHs

For flow cytometry analysis, the single cell populations obtained from dissociated NSPHs were immediately co-stained with a phycoerythrin (PE)-labelled anti-human CD49f antibody (1:20, BD Biosciences, 555736) and a LIVE/DEAD™ Fixable Aqua Dead near-IR Cell Stain (Invitrogen, L34976), according to previously described procedures (33). Flow cytometric analysis was performed using a BD FacsLyric™ analytical flow cytometer (BD Biosciences) and data were analysed using FACSuite v1.5 (BD Biosciences) and FlowJo (10.8.1) software.

### 2.10 Cryosectioning of NSPHs

NSPHs were fixed individually in 1,5 mL of 4% paraformaldehyde solution [PFA in phosphate-buffered saline (PBS)] for 150 min at room temperature (RT). Following two wash steps with 1,5 mL of PBS, NSPHs were stored at 4°C in PBS with 0.01% sodium azide. Following overnight dehydration in 20% sucrose solution (in dH_2_O), NSPHs were embedded in TissueTek-OCT (VWR) for cryosectioning in a NSPH array setup, as previously optimised by us (27). 10 to 20 μm thick frozen sections were prepared using an NX70 cryostar cryostat (Thermo Scientific) and collected on glass slides coated with poly-L-lysine (Sigma) and stored at -20°C before further processing.

### 2.11 Immunofluorescent staining of NSPH cryosections

For immunofluorescence staining, sections were rehydrated with PBS for 5-10 minutes and permeabilised for 30 minutes using 0,1% (v/v) Triton X-100 (Sigma) in Tris-buffered saline (TBS), both at RT. Next, NSPH sections were blocked with a solution of TBS supplemented with 20% serum of the corresponding secondary antibody host species or with 1% bovine serum albumin (BSA) for 1 hour at RT. Next, NSPH sections were incubated overnight at 4°C with primary antibodies diluted either in 10% (m/v) milk solution (Sigma) in TBS or in 3% BSA in TBS. After washing with TBS and a subsequent 1-hour incubation with the secondary antibodies in the dark, slides were washed again and counterstained with DAPI (1 μg/mL, Sigma) for 10 minutes at room temperature. After a final washing step with distilled water, sections were mounted in ProLong R Gold antifade reagent (Thermo Fisher). The used primary and secondary antibodies, as well as their final working concentrations and combinations applied, are provided in **Table 1** (primary antibodies) and **Table 2** (secondary antibodies).

**Table 1:** List of primary antibodies used for immunocytochemistry and flow cytometry.

	Antibody	Host	Source	Final concentration
A	TuJ1	Mouse	R&D Systems (MAB1195)	2 µg/mL
B	MAP2	Chicken	Abcam (ab5392)	1,33 µg/mL
C	NeuN	Guinea pig	Merck Millipore (ABN90P)	1,25 µg/mL
D	GFAP	Rabbit	Abcam (ab7260)	1 - 10 µg/mL
E	S100b	Rabbit	Abcam (ab52642)	5 µg/mL
F	AQP4	Rabbit	Merck Millipore (HPA014784)	3 µg/mL
G	CD49f	Rat	BioLegend (313602)	2 µg/mL
H	CD49f-PE	Rat	BD Biosciences (555736)	NA - 1:20 dilution
I	SOX9	Rabbit	Abcam (ab5535)	0,85µg/mL
J	MX1	Rabbit	Abcam (ab95926)	150 ng/mL
K	IFIT2	Rabbit	Invitrogen (16870404)	0,675 µg/mL
L	ISG15	Rabbit	Proteintech (15981-1-AP)	1,125 µg/mL
M	CD74	Mouse	Invitrogen (15207077)	1,25 µg/mL
N	HLA-DR-PE	Mouse	BioLegend (307605)	NA - 1:200 dilution
O	G3BP1	Mouse	Proteintech (66486-1-lg)	4 µg/mL
P	PABPC	Rabbit	Proteintech (10970-1-AP)	5 µg/mL

NA, not available.

**Table 2:** List of secondary antibodies used for immunocytochemistry.

Antibody	Host	Conjugation	Source	Final concentration	In combination with
Anti-rabbit	Goat	Texas red	Abcam (ab6719)	5 µg/mL	D, E, F, K, L, J
Anti-rat	Goat	AF555	Invitrogen (a21434)	4 µg/mL	G
Anti-mouse	Goat	AF555	Invitrogen (a21425)	2 µg/mL	A, M
Anti-guinea pig	Donkey	Cy3	Jackson ImmunoResearch (706–165-148)	7,5 µg/mL	C
Anti-chicken	Donkey	Cy3	Jackson ImmunoResearch (703-166-155)	1,5 µg/mL	B
Anti-mouse	Goat	AF555	Invitrogen (a21127)	2 µg/mL	O
Anti-rabbit	Goat	AF647	Invitrogen (a21245)	2 µg/mL	P
Anti-rabbit	Goat	FITC	Jackson ImmunoResearch (111-096-114)	7,5 µg/mL	D, I
Anti-chicken	Donkey	FITC	Jackson ImmunoResearch (703-096-155)	3,75 µg/mL	B

### 2.12 Microscopy and image analysis

Live cell images of control and virus infected NSPHs at different time points during culture were captured with a Zeiss Axio Observer.Z1 inverted fluorescence microscope using a N-Achroplan 5x (NA 0,13) objective. Immunofluorescence images of stained NSPHs were acquired using an Olympus BX51 fluorescence microscope equipped with an Olympus DP71 digital camera and using a UPlanFLN 10× (NA 0,30), UPlanFLN 20× (NA 0,50) or PlanC 40× (NA 0,65) dry objective. High-resolution images were acquired using the Nikon CSU-W1 SoRa confocal microscope using a Plan Apo 10x air objective (NA 0.45), Plan Apo 40x air objective (NA 0.95) and Plan Apo 60x water objective (NA 1.2) in normal confocal mode and NIS-Elements software (Nikon). Fiji image analysis freeware was used for image processing and analysis (http://fiji.sc). Briefly, images of stained NSPHs were manually delineated selecting two regions of interest (ROIs): (i) a 200 µm viable border of the NSPH (ROI(i), region of interest), and (ii) the necrotic core of the NSPH (ROI(ii), region of background fluorescence). Results indicating specific immunofluorescence signal of a given marker are presented as mean fluorescence intensity ROI(i)/ROI(ii).

### 2.13 Transmission electron microscopy

VZV^eGFP-ORF23^-infected and control NSPHs were fixed in 2.5% glutaraldehyde in Sorensen’s buffer 0.1 M solution (pH 7.4) at RT for 10 min before being moved 4°C for 2 h. After three washes in Sorensen’s buffer, samples were post-fixed for 60 min in 2% osmium tetroxide and dehydrated through a graded ethanol–propylene oxide series then embedded in epoxy resin. The resin was then polymerised at 60°C for 72 h. Ultrathin Sections (60–80 nm) were cut using a diamond knife (Diatome) mounted in an ultramicrotome (Ultracut S Leica) and contrasted in the dark for 15 min in uranyl acetate solution, and for 15 min in lead citrate solution. For ultrastructural analyses, random fields of these samples were examined under a Jeol TEM JEM-1400 transmission electron microscope at 80 kV, and random fields were photographed using an 11-megapixel camera system (Quemesa, Olympus).

### 2.14 Analysis of cytokine secretion

Concentrations of interleukin 6 (IL-6), CXCL10, interferon alpha-2 (IFN-α2) and interferon beta (IFN-β) in cell culture supernatant were determined using commercially available ELISA MAX™ Deluxe Sets (BioLegend, 430504, 439904, 446404, 449504), according to the manufacturer’s instructions.

### 2.15 Haematoxylin-eosin staining

A haematoxylin and eosin (H&E) staining was performed on cryosections of NSPHs using Carazzi’s haematoxylin (0.1% (m/v) dissolved in 1:4 distilled water:glycerol 85% containing 105 mM KAl(SO_4_)_2_.12 H_2_O, 0.9 mM KIO_3_ Sigma) and eosin Y (1% (m/v) in distilled water, Sigma), as previously described (27). In short, cryosections were stained for 2 min. with Carazzi’s haematoxylin and washed for 5 min. with running water. After 5 min. of eosin Y staining, slides were dipped 5 times in distilled water and subsequently dehydrated by 95% ethanol (2 min.), 100% ethanol (2 min.) and xylene (10 min.) and finally mounted using ProLong^®^ Gold anti-fade reagent (Thermo Fisher).

### 2.16 Nanostring digital transcriptomics

At 7 days post stimulation/infection, NSPHs were washed with ice cold PBS, snap frozen in liquid nitrogen and stored at -80°C. RNA extraction was performed on 2 pooled NSPHs of each condition (three samples per condition) using the RNeasy Mini Kit (Qiagen, 74104) according to the manufacturer’s protocol. RNA concentration was determined with the Qubit RNA Broad Range Assay Kit (Invitrogen, Q10211) and the 260/280 ratio, indicative of RNA purity, was checked using NanoDrop™ 2000/2000c Spectrophotometer (Thermo Scientific, ND-2000). The RNA samples were used for digital transcriptomic analysis (nCounter, NanoString Technologies) on a nCounter^®^ MAX Analysis System, as previously described for other viral infections (34–37). Briefly, RNA extracts were hybridised to ± 600 unique capture/reporter pairs (50bp each) targeting 585 immune transcripts and 15 housekeeping genes, as defined in the Human Immunology V2 nCounter^®^ panel, as well as 6 positive and 8 negative control probes (all from NanoString). Results were sequentially corrected for background (negative control probes), technical variation (positive control probes) and RNA content (housekeeping genes) using nSolver 4.0 (NanoString), followed by differential gene expression (DGE) analysis and gene set enrichment (GSE) analysis (based on GO-terms) using Omics Playground (BigOmics Analytics). The original NanoString digital transcriptomics data are available in the GEO database via accession number GSE273529.

### 2.17 Data representation and statistical analysis

Box/dot plots representing cytokine production and quantification of ICC markers were created with GraphPad Prism v.8.2.1 software. Statistical analyses were carried out using JMP^®^ Pro Version 16 statistical software. All data were modelled using a linear mixed-effects model, accounting for the repeated measures, i.e. independent experiments and/or repeated measurements for each observation. *Post-hoc* analyses for linear mixed-effects models were carried out with Tukey’s HSD correction for multiple comparisons. A p-value <0,05 was considered statistically significant. All other statistical analyses were performed directly within nSolver 4.0 (NanoString) and Omics Playground (BigOmics Analytics) software.

## 3 Results

### 3.1 Longitudinal characterisation of NSPH differentiation

**Figure 1:**
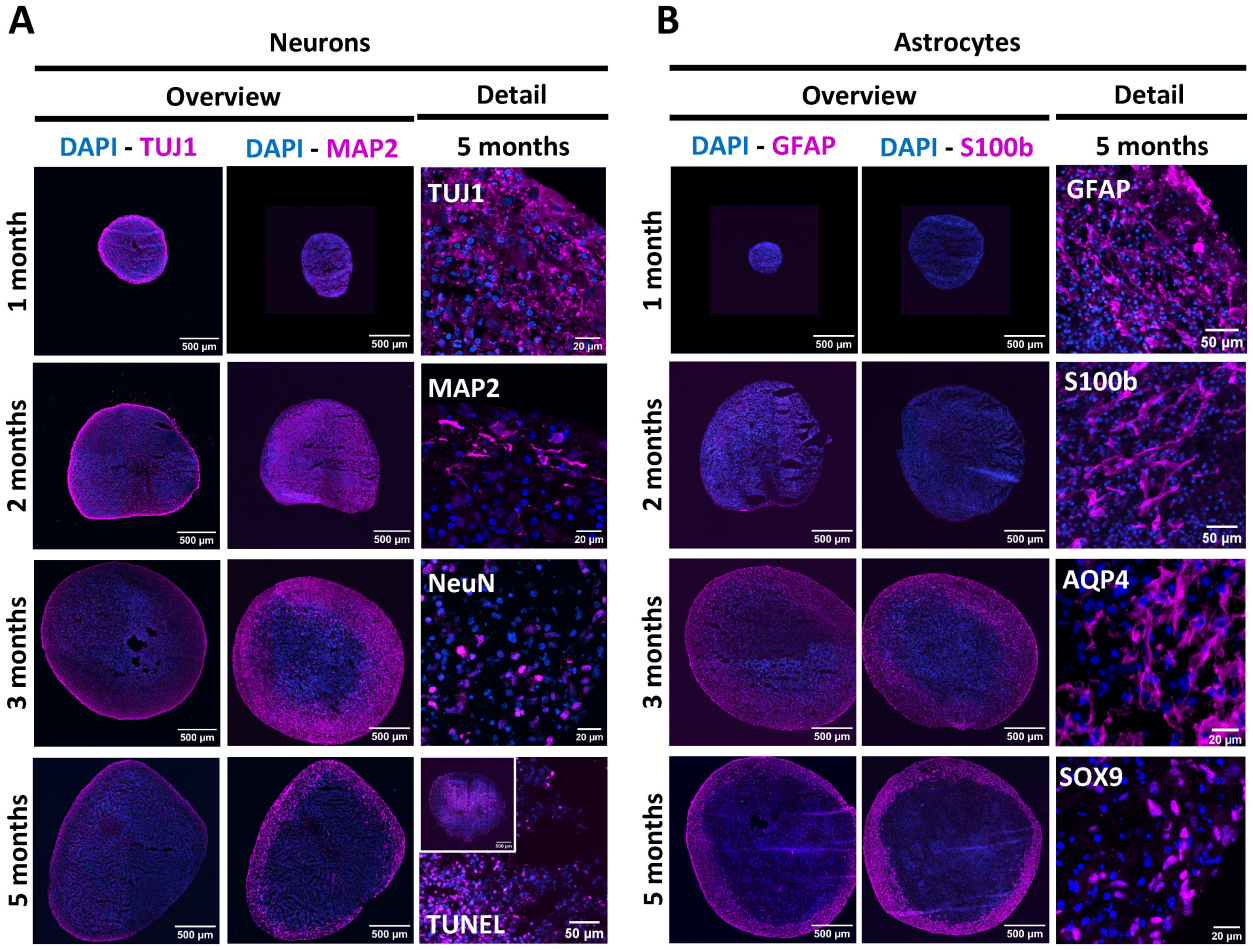
Longitudinal characterisation of NSPH differentiation. **(A)** Representative images of NSPHs at the age of 1, 2, 3 and/or 5 months immunolabelled for the neuronal markers Tuj1 (magenta), MAP2 (magenta) and NeuN (magenta), and the late-stage apoptosis TUNEL staining (magenta), as indicated. **(B)** Representative images of NSPHs at the age of 1, 2, 3 and/or 5 months immunolabelled for the astrocyte markers GFAP (magenta), S100b (magenta), AQP4 (magenta) and SOX9 (magenta), as indicated. Nuclei are labelled with DAPI (blue). Scale bars of 20, 50 and 500 µm are indicated on the images.

To visualise NSPH differentiation longitudinally, NSPHs were harvested after 1, 2, 3 and 5 months of culture and analysed by ICC (**Figure 1**). Following the culture method applied, NSPHs increase in size up to 3 months in culture after which growth stabilised at a diameter of approximately 2-3 mm. Although ICC for neuron-specific proteins (**Figure 1A**) demonstrated the early presence of Tuj1^+^ neurons in 1-month-old NSPHs, expression of the more mature neuronal marker MAP2 was only detected in NSPHs from the age of 2 months. However, due to the increasing size of the NSPHs, a large TUNEL^+^ core of dead cells becomes more pronounced within the NSPHs from 3 months of age onwards. Nevertheless, 5-month-old NSPHs clearly displayed the presence of matured Tuj1^+^MAP2^+^NeuN^+^ neurons in the viable border. In contrast to the early appearance of developing neurons, only a small population of GFAP^+^ astrocytes was detected at earliest after 2 months of culture, and this specifically at the outer border of the NSPH (**Figure 1B**). Upon continued NSPH culture, this immature astrocyte population colonised the viable NSPH border and matured into GFAP^+^S100b^+^AQP4^+^SOX9^+^ astrocytes between the 3^rd^ and the 5^th^ month of age, giving rise to a matured bi-partite (neurons + astrocytes) hiPSC-derived NSPH model that will be used throughout this study.

### 3.2 5-month-old NSPHs are susceptible to VZV^eGFP-ORF23^ infection

**Figure 2:**
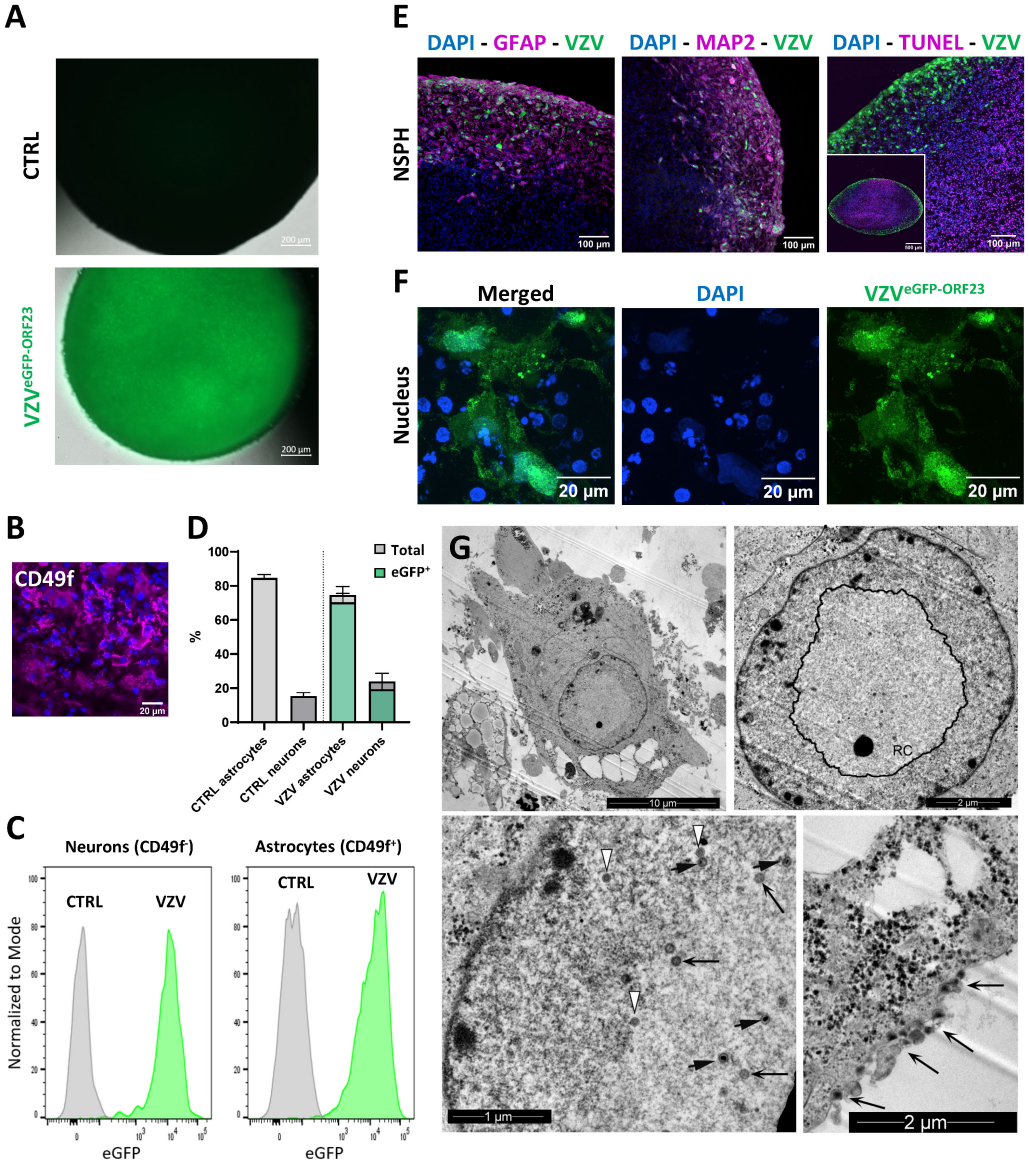
VZV^eGFP-ORF23^ infection of 5-month-old NSPHs. **(A)** Representative live cell fluorescence image of uninfected NSPHs and VZV^eGFP-ORF23^ infected NSPHs at day 7 post-infection. Scale bars of 200 µm are indicated on the images. **(B)** Representative image of a NSPH stained with the extracellular astrocyte-enriched CD49f marker (magenta) and nuclei labelled with DAPI (blue). Scale bar of 20µm indicated on the image. **(C)** Representative flow cytometric analysis showing histogram overlay of CD49f^-^ neurons and CD49f^+^ astrocytes obtained from dissociated uninfected NSPHs (CTRL) and VZV^eGFP-ORF23^ infected NSPHs (VZV) at day 7 post-infection. **(D)** Bar graph indicating the average percentage of astrocytes and neurons measured in uninfected NSPHs (CTRL) and VZV^eGFP-ORF23^ infected NSPHs (VZV), as well as the average % of astrocytes and neurons displaying eGFP-ORF23 fluorescence in VZV^eGFP-ORF23^ infected NSPHs (n=3 for CTRL and n=3 for VZV). Error bars indicate standard deviation (SD). **(E)** Representative images of VZV^eGFP-ORF23^ infected NSPHs (green) at the age of 5 months immunolabelled for GFAP (magenta), MAP2 (magenta) and TUNEL (magenta), as indicated. Nuclei are labelled with DAPI (blue). Scale bars of 100 and 500 µm are indicated on the images. **(F)** Representative images of the cytoplasmic and nuclear localisation of VZV^eGFP-ORF23^ (green) in infected NSPHs at the age of 5 months. Nuclei are labelled with DAPI (blue). Scale bars of 20 µm are indicated on the images. **(G)** Representative transmission electron microscopy (TEM) images of VZV^eGFP-ORF23^ infected NSPHs at the age of 5 months. Upper left image showing an overview TEM image of a VZV^eGFP-ORF23^ infected cell. Upper right image showing the nuclear replication complex (RC). Lower left image showing different maturation stages of the VZV virion, with white arrows indicating procapsids, long black arrows indicating capsid B and short black arrows indicating capsid C Lower right image showing viral particles egressing at the cell surface (long black arrows). Scale bars of 1, 2 and 10 µm are indicated on the images.

At the age of 5 months, NSPHs were inoculated with cell-associated VZV^eGFP-ORF23^, as described in detail in the Materials and Methods section. Following 7 days of infection, widefield live cell fluorescence microscopy revealed a widespread bright eGFP signal from inoculated NSPHs as compared to control NSPHs (**Figure 2A**). Next, flow cytometry of single cell populations dissociated from control and VZV^eGFP-ORF23^-infected NSPHs was performed to determine the proportion of VZV^eGFP-ORF23^-infected astrocytes (CD49f^+^, **Figures 2B, C**) and neurons (CD49f^-/low^, **Figure 2C**) within NSPHs. We found that both the CD49f^+^ cell population (comprising astrocytes) and the CD49f^-^ cell population (comprising neurons) were highly susceptible to VZV^eGFP-ORF23^ infection (**Figure 2C**), with - within the viable cell population recovered - 80,5 +/- 1,3% of neurons and 94,2 +/- 1,2% of astrocytes being infected (**Figure 2D**). Additionally, ICC analysis of cryosections of VZV^eGFP-ORF23^-infected NSPHs (**Figure 2E**; **Supplementary Figure 1**) confirmed widespread eGFP-ORF23 expression throughout the entire viable border of the VZV^eGFP-ORF23^-infected NSPHs, consisting of both astrocytes (GFAP staining) and neurons (MAP2 staining), but not in the necrotic core of the NSPHs (TUNEL staining). Higher magnification confocal images (**Figure 2F**) revealed both nuclear and cytoplasmic localisation of the eGFP-ORF23 fluorescent signal (green), indicative of a productive VZV infection. The latter was further confirmed using TEM, showing the VZV^eGFP-ORF23^ replication complex (RC) in the nucleus (**Figure 2G**, upper left and upper right panel), with the presence of different viral capsid structures (procapsids, capsid B and capsid C) in- and outside of the RC (**Figure 2G**, lower left panel), as well as egressing viral particles at the cell surface (**Figure 2G**, lower right panel). In summary, these results demonstrate the susceptibility of bi-partite hiPSC-derived NSPHs to VZV^eGFP-ORF23^ infection.

### 3.3 Matured NSPHs are immunosensitive, but do not secrete a selected panel of pro-inflammatory cytokines following VZV^eGFP-ORF23^ infection

**Figure 3:**
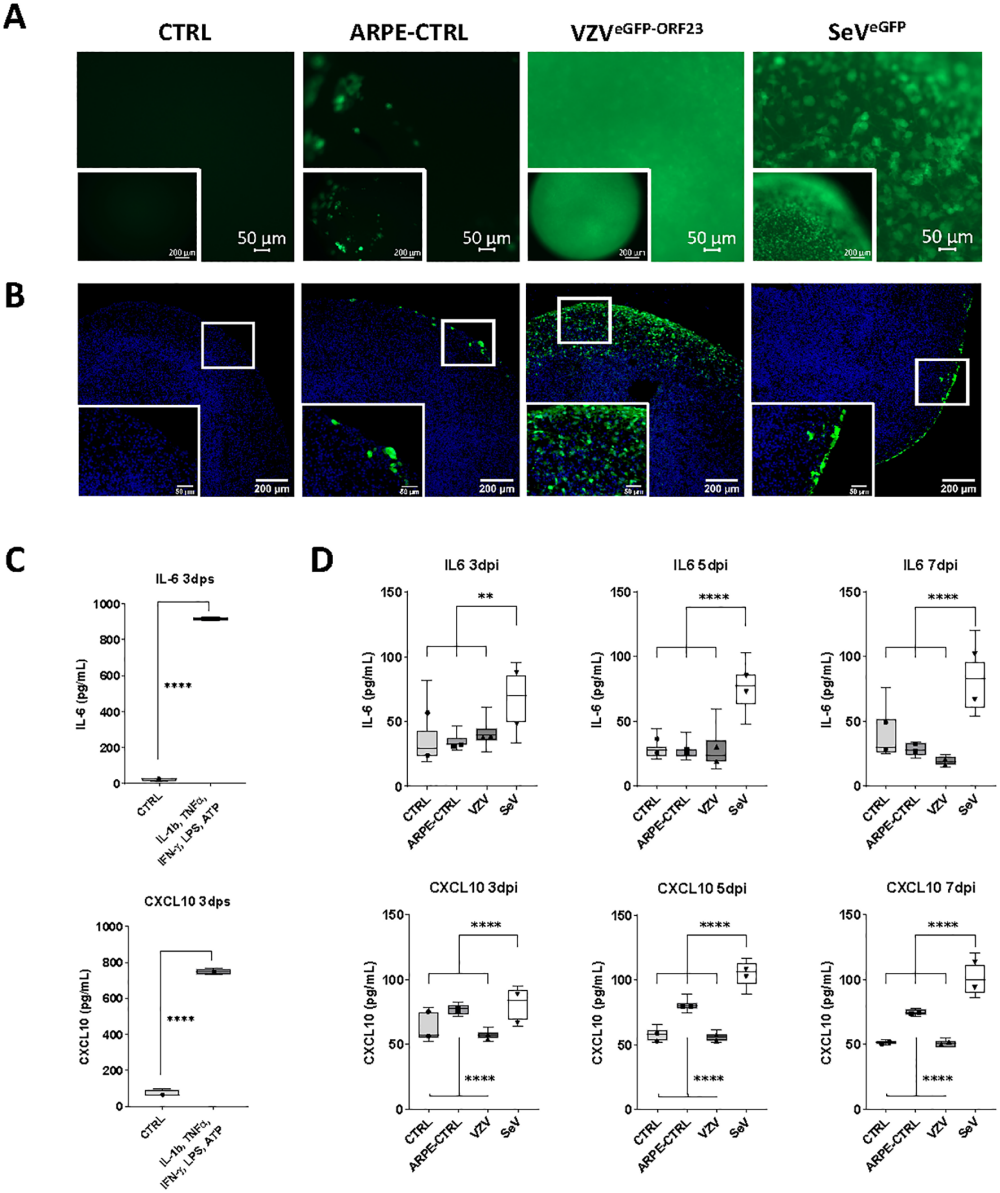
Immune responsiveness of VZV^eGFP-ORF23^- and SeV^eGFP^-infected NSPHs. **(A)** Representative live cell fluorescence image of 5-month-old control NSPHs (CTRL), NSPHs inoculated with eGFP+ control ARPE19 cells (ARPE-CTRL, green), NSPHs inoculated with VZV^eGFP-ORF23^-infected ARPE19 cells (VZV^eGFP-ORF23^, green), and NSPHs infected with SeV^eGFP^ (SeV^eGFP^, green) at day 7. Scale bars of 50 and 200 µm are indicated on the images. **(B)** Representative images of 5-month-old control NSPHs (CTRL), NSPHs inoculated with eGFP+ control ARPE19 cells (ARPE-CTRL, green), NSPHs inoculated with VZV^eGFP-ORF23^-infected ARPE19 cells (VZV^eGFP-ORF23^, green), and NSPHs infected with SeV^eGFP^ (SeV^eGFP^, green) at day 7. Nuclei are labelled with DAPI (blue). Scale bars of 50 and 200 µm are indicated on the images. **(C)** Boxplots showing IL-6 and CXCL10 cytokine secretion (in pg/mL) by 5-month-old control NSPHs (CTRL, n=4) and NSPHs stimulated with IL-1b, TNF-α, IFN-γ, LPS and ATP (n=4) at day 3 post-stimulation (dps). **** p<0,0001. Error bars indicate standard deviation (SD). **(D)** Combined box/dotplots showing IL-6 and CXCL10 cytokine secretion (in pg/mL) derived from 2 independent experiments for5-month-old control NSPHs (CTRL, n=8 for Exp1, n=4 for Exp 2), NSPHs inoculated with eGFP+ control ARPE19 cells (ARPE-CTRL, n=8 for Exp1, n=4 for Exp 2), NSPHs inoculated with VZV^eGFP-ORF23^-infected ARPE19 cells (VZV, n=8 for Exp1, n=4 for Exp 2), and NSPHs infected with SeV^eGFP^ (SeV, n=8 for Exp1, n=4 for Exp 2) at 3-, 5- and 7-days post-infection (dpi). The mean of each individual experiment is given as a dot within the boxplot. ** p<0,01. **** p<0,0001.

Given the high infectivity of NSPHs by VZV^eGFP-ORF23^, and in line with our preceding studies demonstrating the lack of neuronal innate immune signalling towards VZV (5, 25), we here questioned whether astrocytes could immunologically sense the presence of a productive VZV^eGFP-ORF23^ infection. To investigate this, 4 experimental conditions were included: (a) control NSPHs, (b) NSPHs inoculated with eGFP^+^ control ARPE19 cells (= control for condition c), (c) NSPHs inoculated with VZV^eGFP-ORF23^-infected ARPE19 cells, and (d) NSPHs infected with an SeV^eGFP^, serving as a positive control for induction of Type-I/II IFN response. At first, NSPH infectivity with VZV^eGFP-ORF23^ and SeV^eGFP^ was monitored by live cell fluorescence microscopy over 7-days (**Supplementary Figure 2**; **Figure 3A**). This revealed successful NSPH infectivity by VZV^eGFP-ORF23^ and SeV^eGFP^, as demonstrated by a gradual increase in green fluorescent signal. Inoculation of NSPHs with eGFP^+^ control ARPE19 cells only resulted in the appearance of a few eGFP^+^ foci resulting from ARPE19 cells adhering to the NSPHs. Subsequent ICC analysis (**Figure 3B**) however revealed a different infectivity pattern whereby VZV^eGFP-ORF23^ displayed widespread distribution within the viable NSPH border, while SeV^eGFP^ only displayed infectivity in the outer layer of the NSPHs. Next, we investigated the capacity of NSPHs to respond immunologically to VZV^eGFP-ORF23^ or SeV^eGFP^ infection. Although NSPHs secreted high levels of IL-6 and CXCL10 following 3 days of stimulation with a pro-inflammatory cocktail consisting of ATP, LPS, IL-1β, IFNγ and TNFα (**Figure 3C**), no significant release of IL-6 and CXCL10 in the NSPH cell culture supernatant could be detected following VZV^eGFP-ORF23^ infection over the 3 measured timepoints (3-, 5- and 7-days post-stimulation) (**Figure 3D**). In contrast, SeV^eGFP^ infection resulted in a significant release of IL-6 and CXCL10 (**Figure 3D**), albeit at ten-fold lower levels as compared to stimulation with a pro-inflammatory cocktail (**Figure 3C**). Additionally, we investigated the secretion of IFN-α2 and IFN-β at the same timepoints following VZV^eGFP-ORF23^ and SeV^eGFP^ infection. However, for both VZV^eGFP-ORF23^ and SeV^eGFP^, infected NSPHs did not secrete detectable levels of IFN-α2 and IFN-β (data not shown). Concluding, 5-month-old NSPHs are immune responsive to pro-inflammatory stimulation and SeV^eGFP^-infection, as demonstrated by the secretion of IL-6 and CXCL10, but do not secrete pro-inflammatory cytokines following VZV^eGFP-ORF23^-infection, at least not for the panel applied in this study.

### 3.4 Molecular immune profiling of VZV^eGFP-ORF23^and SeV^eGFP^-infected NSPHs

**Figure 4:**
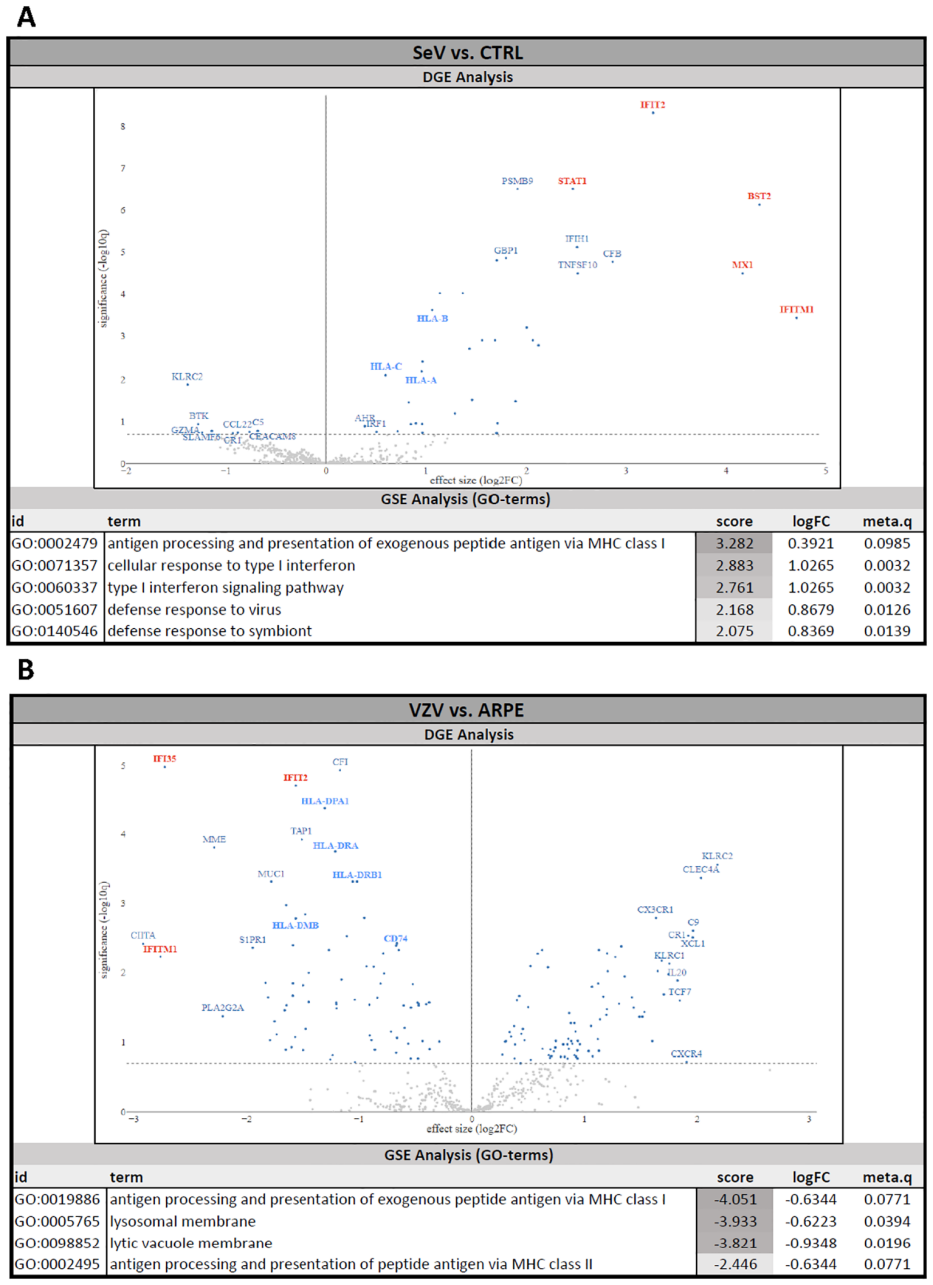
Human Immunology NanoString gene expression analysis of VZV^eGFP-ORF23^- and SeV^eGFP^-infected NSPHs. Differential gene expression (DGE) analysis and gene set enrichment (GSE) analysis. **(A)** NSPHs infected with SeV^eGFP^ (SeV, n=3) vs. uninfected control NSPH (CTRL, n=3). **(B)** NSPHs inoculated with VZV^eGFP-ORF23^-infected ARPE19 cells (VZV, n=3) vs. NSPHs inoculated with eGFP+ control ARPE19 cells (ARPE, n=3). Genes related to the Type-I IFN pathway are marked in red and genes related to the MHC antigen presentation pathway are marked in blue on the DGE volcano plot. The activation score, LogFC and meta.q significance values for the top-ranked GO terms from the GSE analysis are provided for **(A, B)**.

Given the observation that SeV^eGFP^, but not VZV^eGFP-ORF23^, induced cytokine release by infected NSPHs, we performed a multiplex human immunology NanoString gene expression analysis on the 4 experimental conditions described above to gain a broader insight into the immune signalling pathways triggered upon viral infection. DGE analysis between SeV^eGFP^ infected NSPHs and control NSPHs revealed strong upregulation of gene transcripts related to the Type-I interferon response, such as IFIT2, BST2, MX1, IFITM1 and STAT1 (**Figure 4A**, indicated in red on the volcano plot), as well as a moderate upregulation of gene transcripts related to MHC class I antigen presentation, such as HLA-A, HLA-B and HLA-C (**Figure 4A**, indicated in blue on the volcano plot). Consequently, GSE analysis (GO terms) revealed positive enrichment scores (meaning activation) for pathways related to antigen processing and presentation, Type-I IFN signalling and cellular defence against viral infection (**Figure 4A**, GSE table), albeit only the latter two being significant (based on the meta.q value). In contrast, DGE analysis between NSPHs inoculated with VZV^eGFP-ORF23^-infected ARPE19 cells and NSPHs inoculated with eGFP^+^ control ARPE19 cells revealed a significant downregulation of gene transcripts related to the Type-I interferon response, such as IFI35, IFIT2 and IFITM1 (**Figure 4B**, indicated in red on the volcano plot), and gene transcripts related to MHC class II antigen presentation, such as HLA-DPA1, HLA-DRA, HLA-DRB1, HLA-DMB and CD74 (**Figure 4B**, indicated in blue on the volcano plot). Next, GSE analysis (GO terms) revealed negative enrichment scores (meaning suppression) for pathways related to antigen processing and presentation and lysosomal functioning (**Figure 4B**, GSE table), albeit only the latter being significant (based on the meta.q value). Of note, GSE analysis of ARPE19-stimulated NSPHs vs. control NSPHs revealed no significant differences caused by the stimulation of the NSPHs with the ARPE19 vehicle cells (data not shown). Concluding, these results suggest that VZV^eGFP-ORF23^ infection in NSPH, besides not triggering and even actively suppressing a Type-I IFN response, also interferes with antigen processing and presentation processes.

### 3.5 VZV^eGFP-ORF23^ infection of NSPHs interferes with Type-I IFN response and antigen presentation

**Figure 5:**
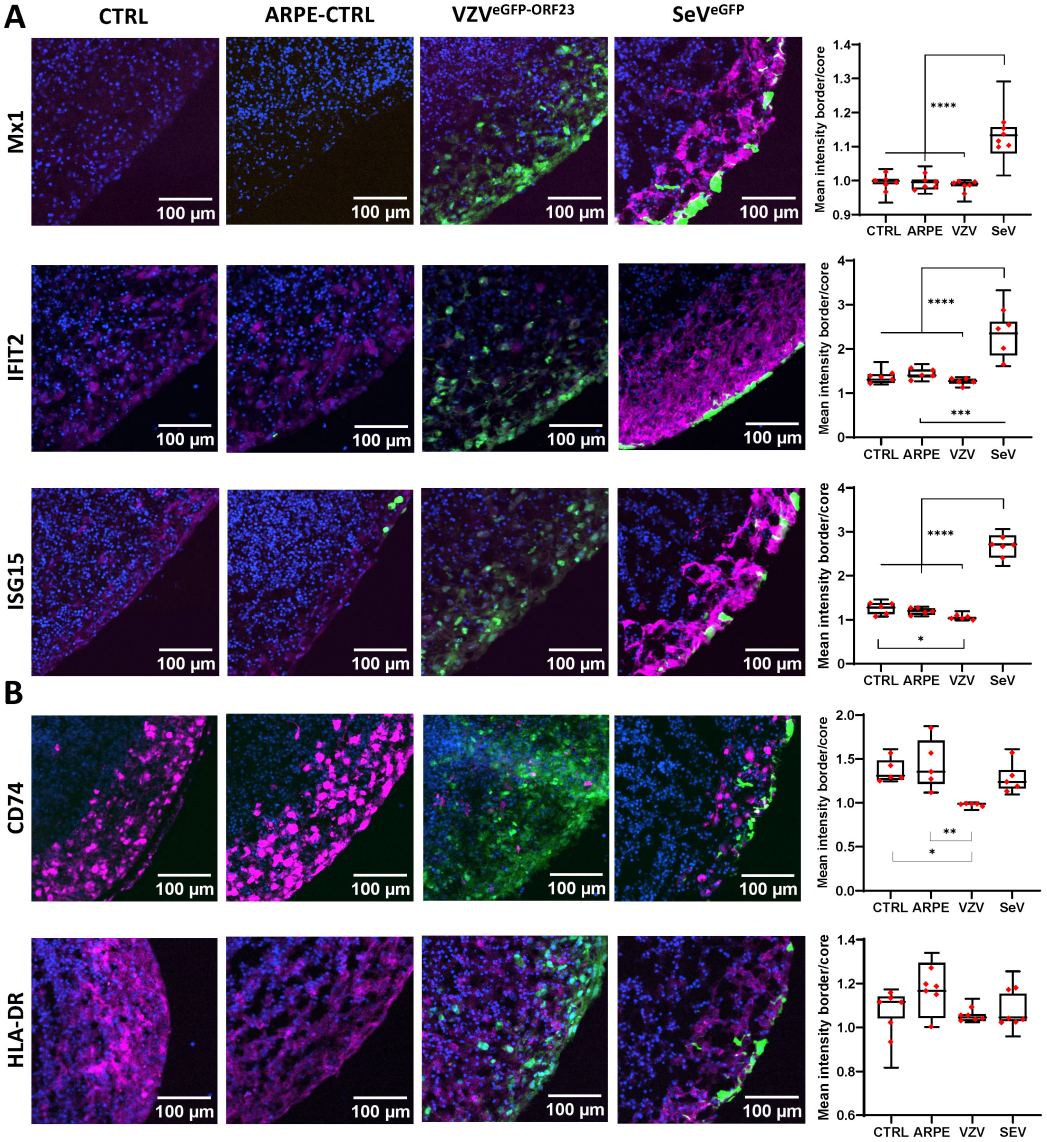
VZV^eGFP-ORF23^ and SeV^eGFP^-infected NSPHs display opposite effects on protein expression related to Type-I interferon response and antigen presentation pathway. Representative immunofluorescence images for 5-month-old control NSPHs (CTRL, n=6), NSPHs inoculated with eGFP+ control ARPE19 cells (ARPE-CTRL, green, n=6), NSPHs inoculated with VZV^eGFP-ORF23^-infected ARPE19 cells (VZV^eGFP-ORF23^, green, n=6), and NSPHs infected with SeV^eGFP^ (SeV^eGFP^, green, n=6), immunolabelled for **(A)** Type-I interferon response markers MX1, IFIT2 and ISG15, and **(B)** antigen presentation pathway markers HLA-DR and CD74. All in magenta. Nuclei are labelled with DAPI (blue). Scale bars of 100 µm are indicated on the images. Combined box/dotplots showing signal quantification. The mean value for each individual NSPH analysed is given as a dot within the boxplot. * p<0,05. ** p<0,01. *** p<0,001. **** p<0,0001.

To validate the observed alterations in molecular signalling pathways, ICC analyses were performed on cryosections obtained under the 4 experimental NSPH conditions described above. First, examination of downstream proteins of the Type-I Interferon pathway (**Supplementary Figure 3**; **Figure 5A**) revealed significant upregulation of MX1, IFIT2 and ISG15 at the protein level in SeV^eGFP^-infected NSPHs, but not in VZV^eGFP-ORF23^-infected NSPHs. Second, investigation of proteins related to MHC Class II antigen processing and presentation (**Supplementary Figure 3**; **Figure 5B**), confirmed downregulation of the invariant chain protein CD74 in VZV^eGFP-ORF23^-infected NSPHs as compared to control NSPHs. However, at this stage of analysis, this did not yet result in a significantly lower expression of HLA-DR. Concluding, these results confirm that VZV^eGFP-ORF23^ interferes with Type-I IFN response and antigen presentation in a human neural-like environment.

### 3.6 VZV^eGFP-ORF23^- and SeV^eGFP^-infection of NSPHs induces stress granule formation

**Figure 6:**
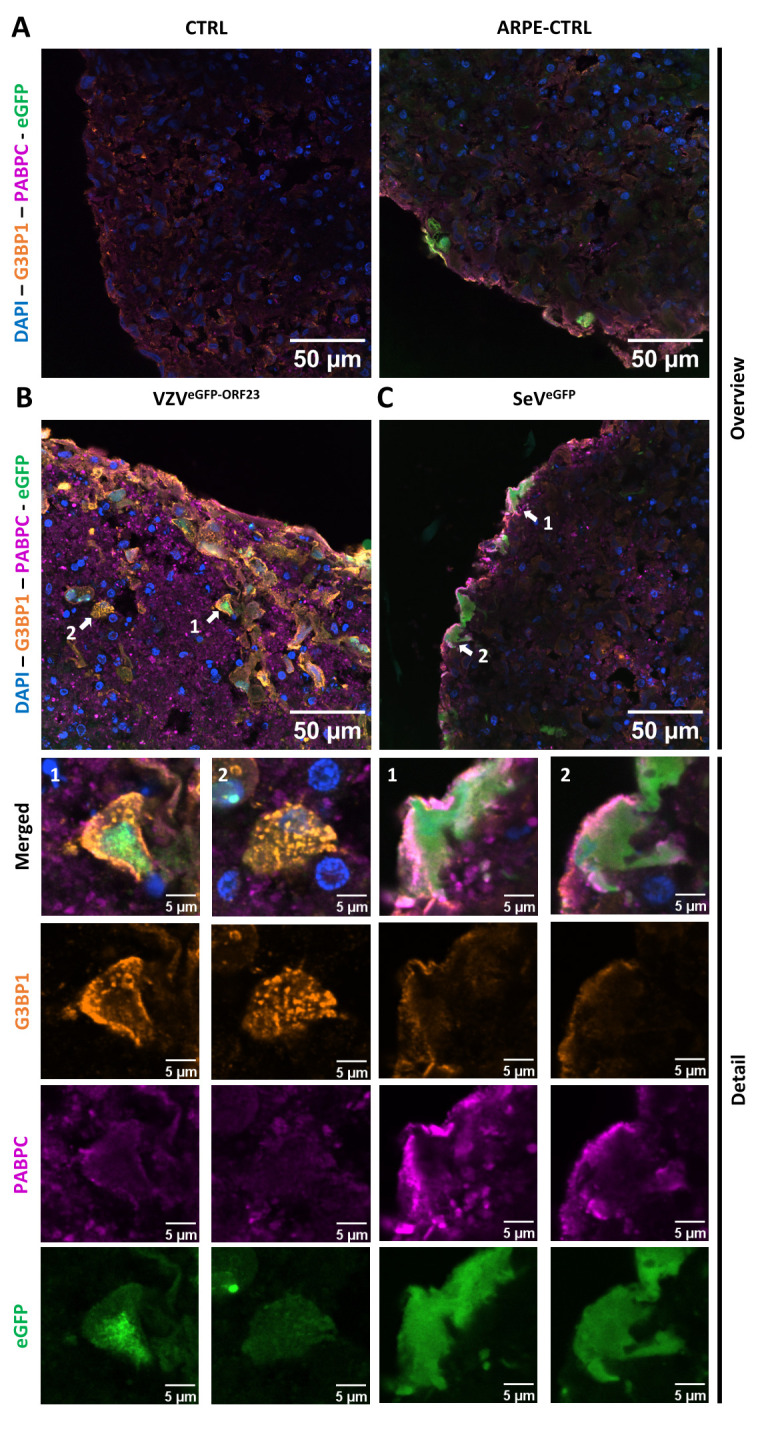
Formation of stress granules in VZV^eGFP-ORF23^ and SeV^eGFP^-infected NSPHs. Representative overview immunofluorescence images of control NSPHs [**(A)**, top left], NSPHs inoculated with eGFP+ control ARPE19 cells [**(A)**, top right, ARPE-CTRL], NSPHs inoculated with VZV^eGFP-ORF23^-infected ARPE19 cells [**(B)**, top left, VZV^eGFP-ORF23^], and NSPHs infected with SeV^eGFP^ [**(C)**, top right, SeV^eGFP^], immunolabelled for G3BP1 (orange) and PABPC1 (magenta). Insets of virus-infected cells are depicted by white arrows on the overview image [**(B, C)**, top] and shown below the corresponding overview images [**(B, C)**, bottom]. Nuclei are labelled with DAPI (blue). Scale bars of 50 (overview) and 5 (detail) µm are indicated on the images.

Even though VZV^eGFP-ORF23^ can evade innate immune signalling in NSPHs, it is highly unlikely that a productive VZV^eGFP-ORF23^ infection has no consequences in infected cells. We questioned whether a cellular stress response was induced in VZV^eGFP-ORF23^- and SeV^eGFP^-infected NSPHs upon prolonged viral challenge of a neural-like environment. Hereto, ICC analyses were performed at 7 days post-infection for 2 well-described protein components of stress granules (SGs), namely G3BP1 (G3BP stress granule assembly factor 1) and PABPC1 (poly(A) binding protein cytoplasmic 1). While no SG formation was observed in control NSPHs and NSPHs inoculated with eGFP^+^ control ARPE19 cells (**Figure 6A**), within VZV^eGFP-ORF23^-infected NSPHs we observed a strong induction of G3BP1^+^ SG formation in VZV^eGFP-ORF23^-infected cells (**Figure 6B**). In contrast, SeV^eGFP^-infected cells in the NSPH induce the formation of PABPC1^+^ SGs, but not G3BP1^+^ SGs, in infected cells (**Figure 6C**). Concluding, these results indicate a clear difference in the type of SGs formed following prolonged viral presence in our NSPH model for both types of viruses. Furthermore, the formation of SGs following VZV^eGFP-ORF23^ infection of the NSPHs is a clear indication of induced cellular stress upon prolonged VZV^eGFP-ORF23^ presence, even though further downstream, VZV still manages to affect innate immune signalling and antigen-processing machinery.

### 3.7 VZV^ORF65-tdT-66^ affects cellular integrity in infected NSPHs

**Figure 7:**
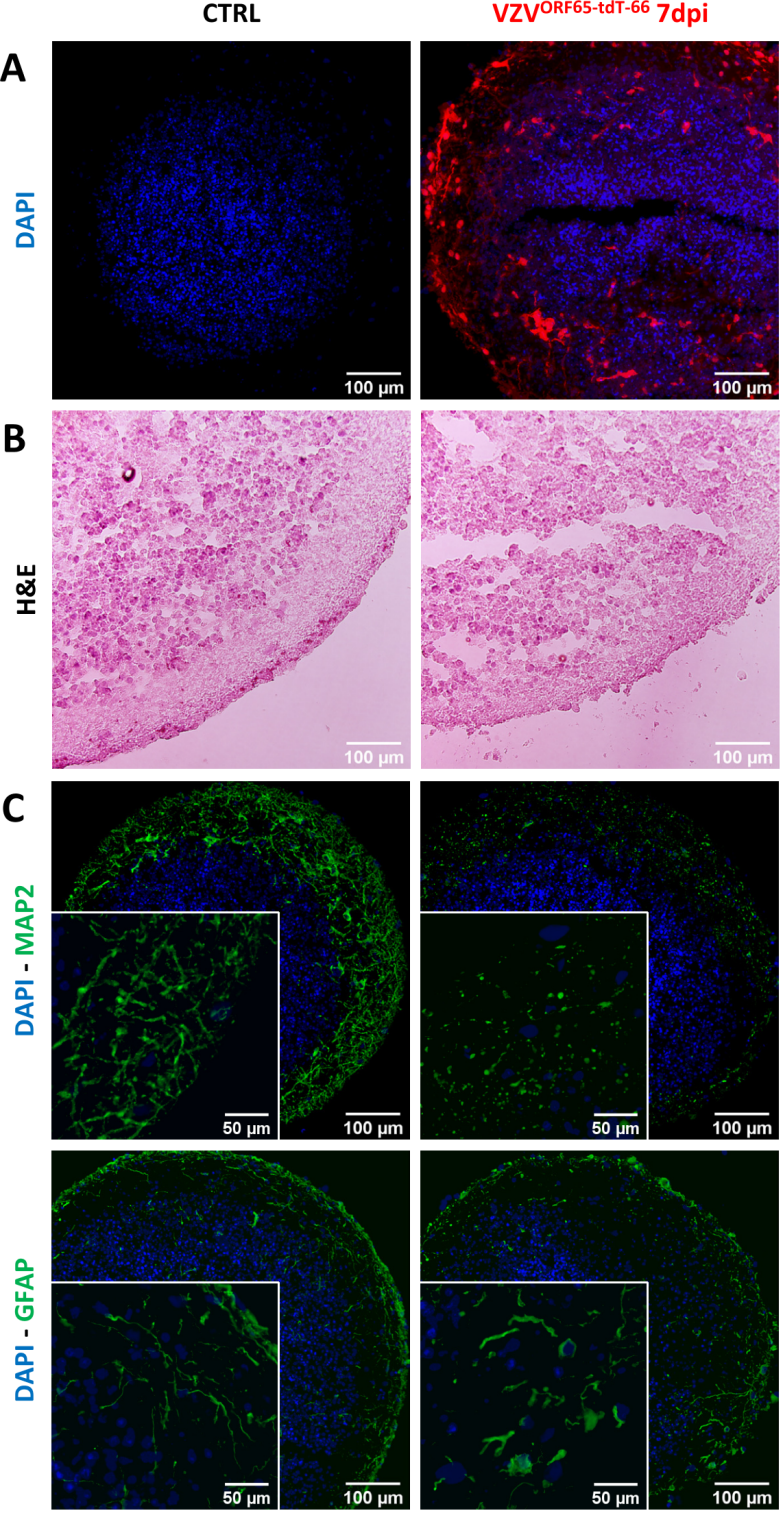
VZV^ORF65-tdT-66^ affects cellular integrity in infected NSPHs. **(A)** Representative images of cryosections of control (CTRL) and VZV^ORF65-tdT-66^ infected NSPH at day 7 post-infection (dpi), in which nuclei are labelled with DAPI (blue) and infection with VZV^ORF65-tdT-66^ is shown in red (direct tdTomato fluorescence signal). **(B)** Representative images Haematoxylin-Eosin stained cryosections of control (CTRL) and VZV^ORF65-tdT-66^ infected NSPH at 7 dpi. **(C)** Representative immunofluorescence images of control (CTRL) and VZV^ORF65-tdT-66^ infected NSPH at 7 dpi, immunolabelled for the neuronal marker MAP2 (green, top panel) and astrocyte marker GFAP (green, bottom panel). Nuclei are labelled with DAPI (blue). Scale bars of 100 (overview) and 50 (detail) µm are indicated on the images.

Further documenting the cellular stress VZV induces in infected NSPHs, we performed a final experiment in which NSPHs were infected with cell-associated VZV^ORF65-tdT-66^ (**Figure 7A**). At first, comparison of Haematoxylin-Eosin (H&E)-stained slides from control and VZV^ORF65-tdT-66^ infected NSPHs already indicates increased structural degradation inside the VZV^ORF65-tdT-66^ infected NSPHs, as well as a clear disruption of the outer border of the NSPH (**Figure 7B**). Although subject to further investigation, additional stainings for the neuronal marker MAP2 and the astrocyte marker GFAP indicate that the morphology and/or integrity of both NSPH cell types is severely affected following infection with VZV^ORF65-tdT-66^ (**Figure 7C**).

## 4 Discussion

While several human pluripotent stem cell (PSC)-derived *in vitro* models have been developed and successfully applied to demonstrate neuronal susceptibility to VZV infection, and to investigate subsequent downstream cell- and/or virus-specific intracellular events (5, 21–25), to date little is known about the behaviour of VZV in a multicellular context, especially including cell types involved in innate immunity (38). This approach is highly important as during primary infection, as well as during secondary infection following reactivation from latency, innate immune signalling is the first line of VZV’s recognition by the host’s immune system that can be activated either by the infected cell itself or by surrounding bystander cells (39–41). In the CNS, besides microglia, astrocytes are in close contact with neurons too, and as such most likely one of the first cells to come into contact with viral particles released from infected neurons (42–45). As mentioned before, there are very few studies concerning VZV neuro-immune biology in primary human brain tissue. A preceding study by Bubak and colleagues has already shown that VZV alters morphology and suppresses pro-inflammatory cytokines in primary human spinal cord and hippocampal astrocytes during VZV infection *in vitro*, and these events may as such represent an immune evasion strategy during VZV myelopathy and encephalopathy (46). While this study already provided valuable insights into (the absence of) an anti-viral astrocyte response upon VZV challenge, the use of human post-mortem isolated and cultured astrocytes may not fully recapitulate *in vivo* physiological responses due to the absence of important astrocyte-neuron interactions.

Mixed human neuron-astrocyte cultures are difficult to establish in a 2D context due to differences in growth factor requirements for both cell types and the time needed to regain physiological rest and functional maturity upon plating of individual cell populations (11, 47). For this reason, PSC-derived NSPHs have gained significant importance in the study of virus infections of the CNS as they have been able to overcome some of the major limitations of the preceding preclinical models (7, 8, 19, 48–50). Nevertheless, Depla and colleagues recently pointed out that heterogeneity in organoid generation protocols, as well as their age/maturation at the time of infection, may explain confounding results (7). With a specific focus on studying astrocyte-mediated anti-viral immune responses, it is of utmost importance to allow for time-dependent maturation of the astrocyte-lineage cells in human NSPHs from an embryonic-like developmental stage to a stage resembling at least late-foetal/early-postnatal or even adult human astrocytes, which requires a prolonged maturation period (9, 11). Based on these studies by Sloan et al. (11) and Gordon et al. (9), we decided to define the appearance of AQP4 as a marker for mature(d) adult-like astrocytes, which appeared in this study - with the hiPSC-line and culture protocol used - highly expressed in 5-month-old NSPHs. Therefore, in this study NSPHs were allowed to differentiate/mature for 5 months after which they displayed a stable composition of neurons and astrocytes expressing a clear set of markers associated with matured neurons and astrocytes (**Figure 1**) (9, 11, 31, 51). Furthermore, immune-competence of our cultured NSPHs was demonstrated by release of the pro-inflammatory cytokines IL-6 and CXCL10 following pro-inflammatory cocktail stimulation, previously defined by us (52) and others (31, 46, 53, 54) to demonstrate astrocyte reactivity.

Upon initial VZV^eGFP-ORF23^ infection experiments of 5-month matured NSPHs, we observed VZV^eGFP-ORF23^ spreading rapidly throughout the viable area of the NSPHs, infecting both neurons and astrocytes (**Figure 2**). In agreement with the preceding study by Bubak et al. (46), we here demonstrate that immune-competent NSPHs, with their immune-responsiveness being demonstrated by cytokine production following pro-inflammatory cocktail stimulation or following SeV^eGFP^-infection, are not triggered to release the pro-inflammatory cytokines IL-6 and CXCL10 upon VZV ^eGFP-ORF23^-infection (**Figure 3**). These observations in our 5-month matured NSPH model, wherein we initially hypothesised VZV immune recognition to be coordinated by astrocytes, thus recapitulate the findings of Bubak et al. (46) that also astrocytes – even within a more neural-like context - become subjected to the extensive repertoire of immune evasion strategies of VZV (1, 55–57). A major advantage of this NSPH approach – in contrast to scarce human material – is the (theoretically) unlimited amount of neural-like tissue that can be generated and used for research purposes. As shown in this study, a multiplex human immunology NanoString gene expression analysis (**Figure 4**), and subsequent histological confirmation (**Figure 5**), could easily be performed and demonstrated the ability of VZV^eGFP-ORF23^ to interfere with Type-I interferon response as well as antigen presentation pathways in matured hiPSC-derived NSPHs. Even though this was highly expected based on published literature reviews regarding HSV and VZV immunobiology (1, 38, 58–62), the multicellular context of hiPSC-derived NSPH models allows for single model validation strategies in a human neural-like context.

Noteworthy, next to IL-6 and CXCL10, we also investigated whether NSPHs could be triggered to release IFN-α2 and IFN-β upon infection with VZV^eGFP-ORF23^ or SeV^eGFP^, as a sign of intrinsic anti-viral response. Our observation that no Type-1 IFNs were detected in the culture supernatant in VZV^eGFP-ORF23^ and SeV^eGFP^ infected NSPHs at day 3, day 5 or day 7 post-infection, is in fact also in line with our multiplex human immunology NanoString gene expression analysis, whereby no significant upregulation of IFN-α2 and IFN-β mRNA was noted (**Supplementary Table 1**). Although not yet investigated in detail by us for hiPSC-derived astrocytes and/or NSPHs as a whole, we did report previously that hiPSC-derived neurons, even though they can respond to exogenous IFN-α, they cannot produce IFN-α themselves even after stimulation with strong synthetic inducers (25). Based on literature reports, we hypothesised this to be an intrinsic defence mechanism of the brain against IFN-α mediated neuronal damage. As such, it is also not unexpected that no IFN-α2 and IFN-β was detected upon SeV^eGFP^ infection of NSPHs.

An interesting finding from our approach relates to the invariant chain protein CD74, which – besides stabilising newly generated MHC class II molecules - promotes the intracellular trafficking of empty MHC class II proteins from the endoplasmic reticulum (ER) via the Golgi Apparatus towards late-stage phagolysosomes for subsequent MHC class II antigen-loading (38). Although interference with CD74 has been observed for HSV-1 (38, 63), our new study now also ascribes this immune evasion mechanism also to VZV. In addition, although not further investigated in this study, our GSE analysis (**Figure 4**) also revealed a significant downregulation of signalling pathways related to lysosomal membranes in VZV^eGFP-ORF23^-infected NSPHs. Indeed, several herpesviruses have developed strategies to escape autolysosomal degradation, although there is an increasing body of evidence suggesting complex and sometimes opposing effects of autophagy in the context of VZV infection (64–66), with one recent study suggesting a neuro-protective role for autophagy in VZV-infected neurons (67). Clearly, this is a topic requiring further research.

Regarding the methodological level of NSPH research, we acknowledge several improvements that ultimately need to be integrated, one of which is the inclusion of autologous macrophages and/or microglia (68). Hereto, several protocols have been developed to generate hiPSC-derived NSPH models containing isogenic microglia (69, 70). However, based on our preceding research whereby isogenic hiPSC-derived macrophages were added in co-culture with hiPSC-derived peripheral nervous system (PNS)-like neurons (5), we do not expect them to play a direct role in counteracting a productive VZV-infection, not in the PNS nor in the CNS. As hypothesised before, macrophages/microglia, and potentially even astrocytes, may play an important role in linking innate and adaptive immunity. We suggest that, both in PNS and CNS, respectively macrophages and microglia/astrocytes, will be subject to inhibition of Type-I IFN signalling, but may still play an important role, albeit (partially) suppressed, in primary/secondary T-cell activation (5, 38, 71, 72). As shown in this study, the antigen-presentation machinery becomes subject to downregulation by VZV^eGFP-ORF23^ infection but does not seem to be fully absent as it may require a certain time to fully downregulate HLA-DR expression (**Figures 4**, **5**). To investigate the latter, it would be highly interesting to generate iPSC-lines from both primed (naturally or vaccinated) and naïve healthy individuals, whereupon VZV-infected hiPSC-derived NSPHs can be co-cultured with autologous peripheral blood mononuclear cells (PBMC). In this context, we have recently demonstrated that not only a lower VZV-specific T-cell receptor (TCR) diversity, but also reduced functional TCR affinity for VZV-specific proteins in HZ patients, leads to lower T cell activation and consequently affects the susceptibility for viral reactivation (73). While such studies are currently not feasible due to financial constraints, it is plausible that reduced TCR diversity and/or affinity may aid to susceptibility for VZV-induced CNS neuropathology. Likewise, with the knowledge that several mutations are known to be involved in increased VZV-associated neuropathology (41, 74), in time the use of patient-derived iPSC and autologous PBMC may need to be explored to gain a better understanding of VZV-associated neuropathology and/or potential neuroprotective interventions.

Still under continuous development by many research groups, another limitation of most of the current NSPH models, including ours, is the lack of vascularisation. While on one hand micro-vascularisation may prevent the occurrence of TUNEL^+^ necrotic cells in the NSPH core (**Figure 1**) (75, 76), in the context of VZV-associated neuropathology it may more readily be considered as a way to model VZV vasculopathy and subsequent CNS infection. Specifically for modelling the latter, one may not need a fully vascularised and perfused NSPH model, but rather an external endothelial cell layer, similar to existing blood-brain-barrier (BBB) models (77). Again, these approaches may need to be performed in an autologous experimental setup, as discussed above.

Nevertheless, we would like to emphasise the future importance of multicellular NSPH models, especially as they allow the study of cellular stress in a 3D neural-like context. This is exemplified by our staining for SGs, a stress-induced membraneless organelle, in which over 140 cytoplasmic proteins can intertwine with cytoplasmic mRNAs, translation initiation components and proteins affecting mRNA function (78, 79). While the initial stage of SG formation is considered to be protective, as they allow the cell to control energy consumption in favour of cell survival, their long-term persistence and/or reduced clearance may lead to the activation of cell death processes, as is the case in the pathogenesis of many neurodegenerative diseases (80). In this study, we observed a marked increase in G3BP1^+^ cytoplasmatic aggregates in VZV^eGFP-ORF23^-infected NSPHs (**Figure 6**), suggesting active cellular stress upon sustained NSPH infection (i.e. 7 days post-infection). Although further comparative studies between HSV- and VZV-infections in NSPHs are required, the induced formation of G3BP1+ SGs as such is a novel finding for VZV and has not previously been observed for wild-type HSV (81), although this may be cell-type and/or context dependent, and more specifically in our study the chosen (late) timing of analysis following initial infection. Finnen and colleagues did observe G3BP1+ SGs in cell lines infected with an engineered mutant HSV-2 strain, however, these SGs were not observed following infection with wild-type HSV-2 (82). On the other hand, we observed induction of PABPC1+ SG formation in SeV^eGFP^-infected NSPHs (**Figure 6**), highlighting a clear difference between the two viral infections in terms of the types of SGs induced in the NSPH model. In contrast to VZV, no G3BP1+ SG formation was observed in the SeV^eGFP^-infected NSPHs, which may be consistent with previous studies by Iseni and colleagues indicating that SeV inhibits SG formation by investigating another SG component, TIAR1, which was not tested in our case (81, 83). Like TIAR, G3BP1 is also a primary SG nucleating protein involved in phase 2 of SG assembly (81), further suggesting interference by SeV during SG formation. Although not the initial aim of this study, our NSPH model may thus become an interesting future tool to study SG formation and/or resolution in a more complex *in vitro* human neural-like environment upon viral infection and/or other neuropathologies.

## 5 Conclusion

In this study we have demonstrated that matured 5-month-old hiPSC-derived NSPHs, containing neurons and astrocytes, are immune competent, susceptible to viral infection, and most importantly, able to recapitulate VZV- and SeV-specific innate immune signatures. In this model, we demonstrate that VZV evades innate neuro-immune recognition by suppressing the Type-I IFN and antigen presentation pathways, in contrast to SeV. Furthermore, even though VZV is highly immune evasive, NSPHs do suffer from long-term cellular stress upon infection. This NSPH model is therefore well suited to study viral neuro-immune responses and evasion strategies in a human CNS-like environment.

## Data Availability

The datasets presented in this study can be found in online repositories. The names of the repository/repositories and accession number(s) can be found below: GSE273529 (GEO).

## Supplementary material

Supplementary Figure 1 Single channel images of **Figure 2E**. Representative images of 5-month-old VZV^eGFP-ORF23^ infected NSPHs as seen in **Figure 2E**, but depicted individually as single-channel images of the nuclei labelled with DAPI (blue), the VZV^eGFP-ORF23^ infection (green) and the stainings (magenta). Scale bars of 100 µm are indicated on the images.

Supplementary Figure 2 Longitudinal imaging of VZV^eGFP-ORF23^ and SeV^eGFP^ infection in NSPHs. Representative live cell fluorescence image of 5-month-old control NSPHs (CTRL), NSPHs inoculated with eGFP+ control ARPE19 cells (ARPE-CTRL, green), NSPHs inoculated with VZV^eGFP-ORF23^-infected ARPE19 cells (VZV^eGFP-ORF23^, green), and NSPHs infected with SeV^eGFP^ (SeV^eGFP^, green) at day 3, 5 and 7. Scale bars of 50 and 200 µm are indicated on the images.

Supplementary Figure 3 VZV^eGFP-ORF23^- and SeV^eGFP^-infected NSPHs display opposite effects on protein expression related to Type-I interferon response and antigen presentation pathway – Overview images. Representative images for 5-month-old control NSPHs (CTRL), NSPHs inoculated with eGFP+ control ARPE19 cells (ARPE-CTRL, green), NSPHs inoculated with VZV^eGFP-ORF23^-infected ARPE19 cells (VZV^eGFP-ORF23^, green), and NSPHs infected with SeV^eGFP^ (SeV^eGFP^, green), immunolabelled for the Type-I interferon response markers MX1, IFIT2 and ISG15, and the antigen presentation pathway markers HLA-DR and CD74. All in magenta. Nuclei are labelled with DAPI (blue). Scale bars of 500 µm are indicated on the images.

Supplementary Table 1 Complete gene expression list Human Immunology V2 nCounter^®^ panel. Gene expression list showing 585 immune transcripts, 15 housekeeping genes, 6 positive and 9 negative control probes for the 4 conditions ‘CTRL’, ‘ARPE-GFP’, ‘VZV^eGFP-ORF23^’ and ‘SeV^eGFP^’. The included gene expression data was sequentially corrected for background, technical variation and RNA content, as described in M&M section 2.16.
