# Supplementary figures and images for: Varicella-zoster virus recapitulates its immune evasive behaviour in matured hiPSC-derived neurospheroids
Source: Front Immunol. 2024 Sep 16;15:1458967. doi: 10.3389/fimmu.2024.1458967 (PMC11439716; doi:10.3389/fimmu.2024.1458967)

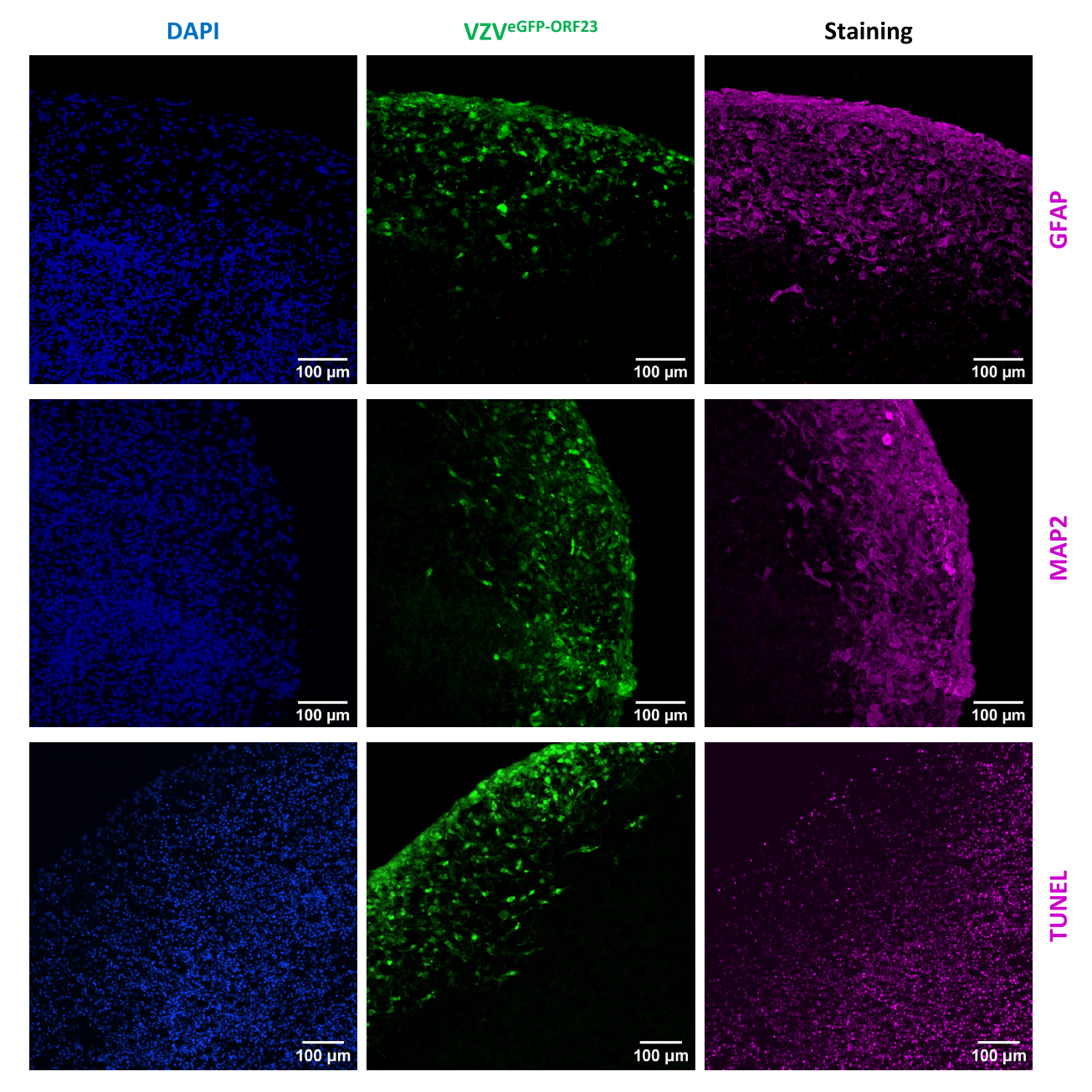

Supplement: Supplementary Figure 1 — Single channel images of Figure 2E . Representative images of 5-month-old VZVeGFP-ORF23 infected NSPHs as seen in Figure 2E , but depicted individually as single-channel images of the nuclei labelled with DAPI (blue), the VZVeGFP-ORF23 infection (green) and the stainings (magenta). Scale bars of 100 µm are indicated on the images. [file Image1.tif]

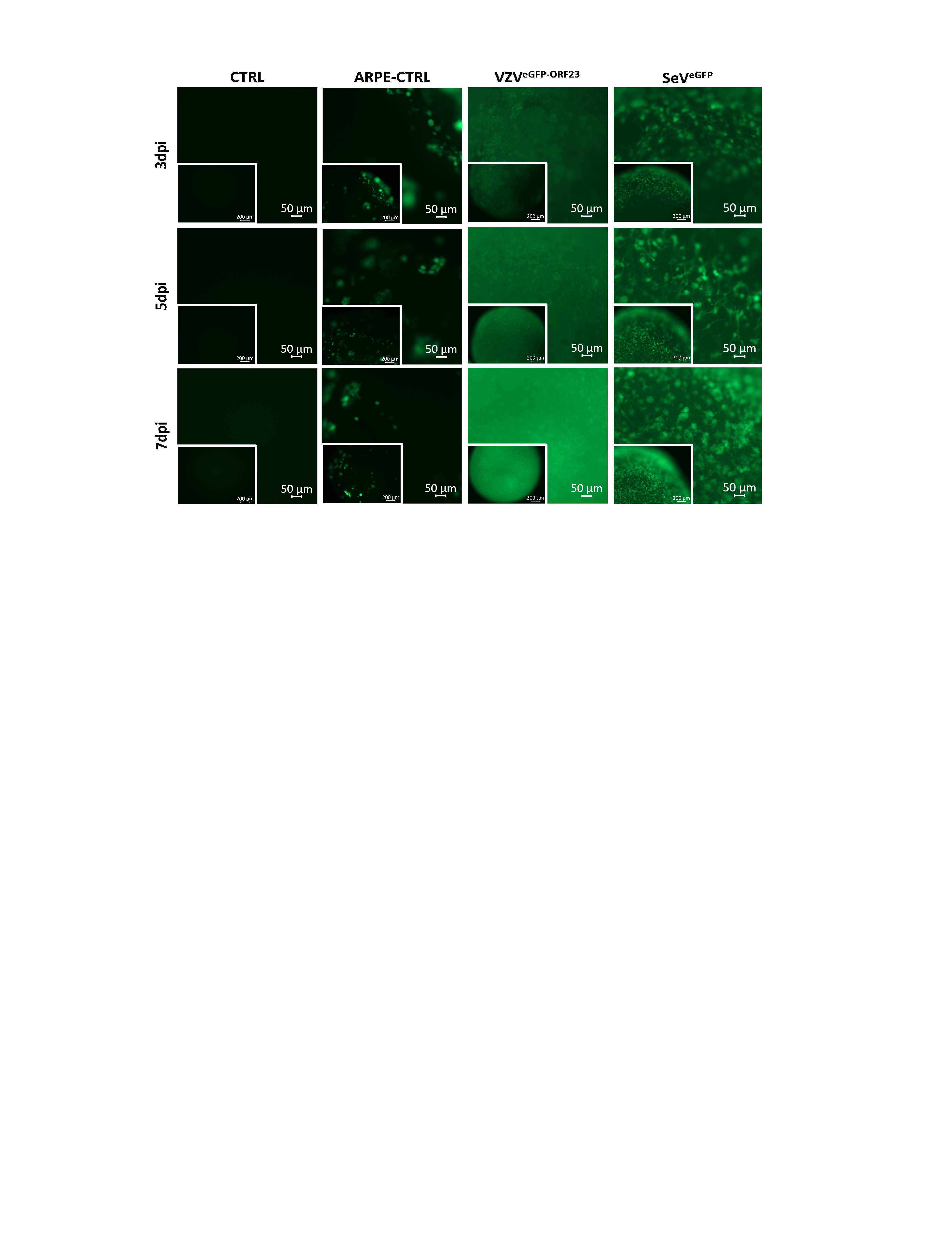

Supplement: Supplementary Figure 2 — Longitudinal imaging of VZVeGFP-ORF23 and SeVeGFP infection in NSPHs. Representative live cell fluorescence image of 5-month-old control NSPHs (CTRL), NSPHs inoculated with eGFP+ control ARPE19 cells (ARPE-CTRL, green), NSPHs inoculated with VZVeGFP-ORF23-infected ARPE19 cells (VZVeGFP-ORF23, green), and NSPHs infected with SeVeGFP (SeVeGFP, green) at day 3, 5 and 7. Scale bars of 50 and 200 µm are indicated on the images. [file Image2.tiff]

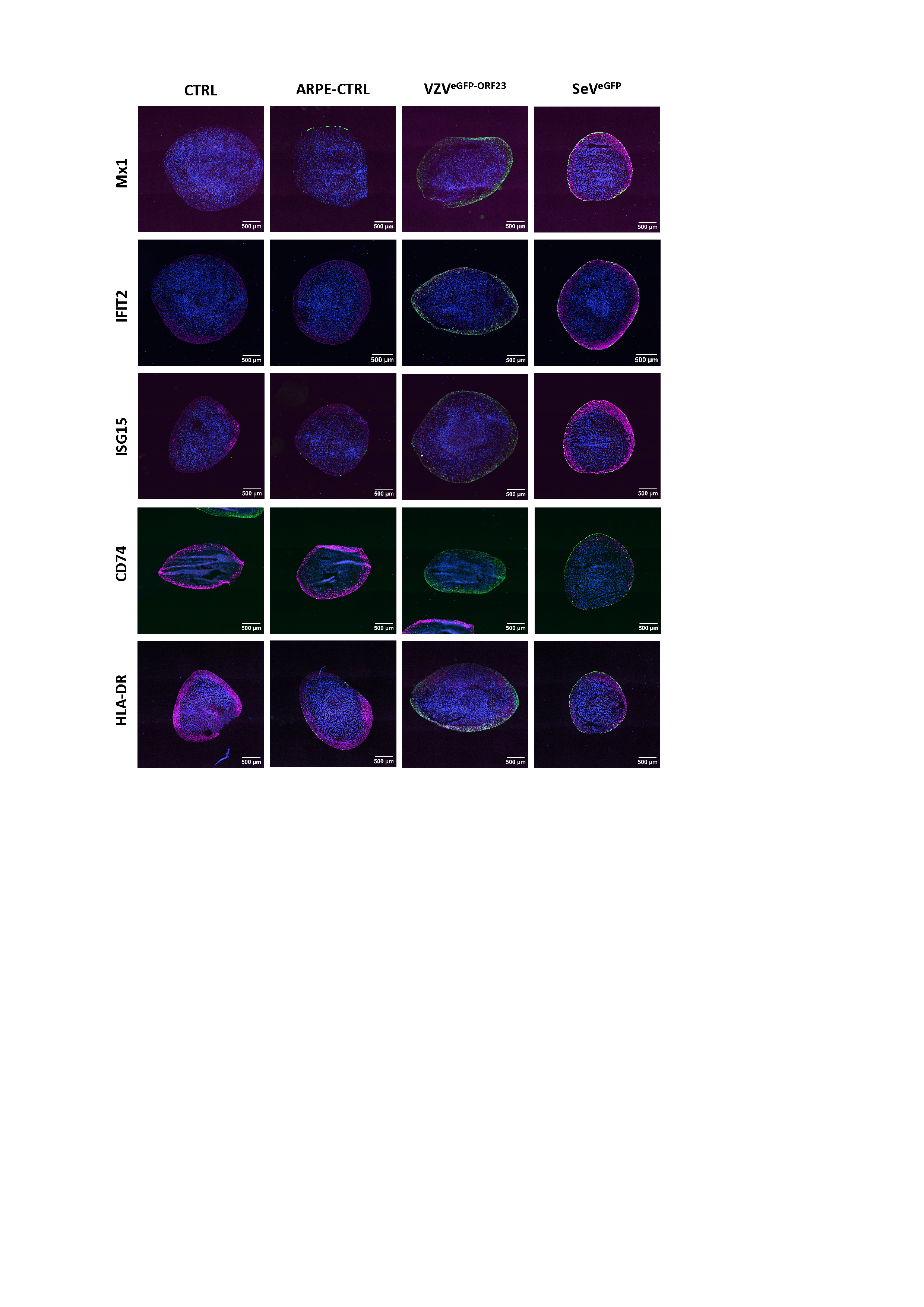

Supplement: Supplementary Figure 3 — VZVeGFP-ORF23- and SeVeGFP-infected NSPHs display opposite effects on protein expression related to Type-I interferon response and antigen presentation pathway – Overview images. Representative images for 5-month-old control NSPHs (CTRL), NSPHs inoculated with eGFP+ control ARPE19 cells (ARPE-CTRL, green), NSPHs inoculated with VZVeGFP-ORF23-infected ARPE19 cells (VZVeGFP-ORF23, green), and NSPHs infected with SeVeGFP (SeVeGFP, green), immunolabelled for the Type-I interferon response markers MX1, IFIT2 and ISG15, and the antigen presentation pathway markers HLA-DR and CD74. All in magenta. Nuclei are labelled with DAPI (blue). Scale bars of 500 µm are indicated on the images. [file Image3.tif]
